# Beamforming Techniques for Over-the-Air Computation in MIMO IoT Networks

**DOI:** 10.3390/s20226464

**Published:** 2020-11-12

**Authors:** Young-Seok Lee, Ki-Hun Lee, Bang Chul Jung

**Affiliations:** Department of Electronics Engineering, Chungnam National University, Daejeon 34134, Korea; yslee@o.cnu.ac.kr (Y.-S.L.); kihun.h.lee@cnu.ac.kr (K.-H.L.)

**Keywords:** over-the-air computation (AirComp), Internet-of-things (IoT) networks, multiple-input multiple-output (MIMO), beamforming, maximum-ratio transmission (MRT)

## Abstract

In this paper, a novel beamforming technique is proposed as the over-the-air computation (AirComp) framework in a multiple-input multiple-output (MIMO) Internet-of-things (IoT) network consisting of multiple IoT sensors (STAs) and a single access point (AP). We assume that each IoT device has the channel state information (CSI) from itself to the AP and the AP has the global CSI of all IoT devices. We consider the mean squared error (MSE), which represents the reliability of function computation, as a performance metric. In short, each IoT device exploits maximum-ratio transmission (MRT) as a transmit beamforming technique to improve MSE performance by taking full advantage of multiple transmit antennae. Moreover, for the receive beamforming, we first consider a receive antenna selection (RAS) technique as the simplest beamforming method at the AP. Then, a semi-definite relaxation (SDR) method and a successive convex approximation (SCA) algorithm are considered and compared with each other in terms of MSE. Finally, we propose a novel two-step beamforming algorithm to further improve the MSE performance of the aforementioned techniques. We have numerically verified through computer simulations that the proposed framework has an improved MSE performance of about 6dB compared to the conventional single-input multiple-output (SIMO) AirComp, even with only two transmit antennae, and the modified MRT outperforms the other transmit beamforming techniques. Furthermore, the proposed receive beamforming technique, a two-step algorithm, shows the best performance in terms of MSE compared to prior studies.

## 1. Introduction

With the skyrocketing growth in the number of devices connected to the Internet, such as the Internet-of-things (IoT) sensors, the International Data Corporation (IDC) forecasts that there will be 41.6 billion connected IoT devices generating 79.4 zettabytes (ZB) of data traffic in 2025 [1]. From this perspective, a technique that can efficiently address distributed IoT sensor data is required. Indeed, one of the main purposes of the IoT sensor network is to compute some objective functions such as average, sum, maximum, minimum, etc. This means that, in some cases, the networks only require the result of the function computation rather than the detection of each sensor datum. For example, when measuring the average temperature in an area, the application will be interested in the computation result, not the temperature value measured by each sensor. In conventional IoT sensor networks, the access point (AP) must detect the readings of each IoT sensor to compute the objective function, i.e., the “transmit-then-compute” process is performed. However, in future massive IoT sensor networks, this manner will lead to problems of excessive latency and radio resource scarcity problems [2,3].

Over-the-air computation (AirComp) is known as a technique that can overcome these issues by computing the objective function of the network without an independent detection process for readings of each IoT sensor. Specifically, the AirComp technique exploits the superposition property of the multiple access channel (MAC). Each sensor simultaneously transmits its reading to an AP through the same sub-carrier. Then, the AP receives the superposed signal weighted by the wireless channel gain. At this time, it can be seen that if each weight, i.e., wireless channel gain, is one and the noise is ignored, the summation result of the readings of each sensor can be obtained. For this process, some pre- and post-processing functions have been introduced as the nomographic form [4,5]. Although pre- and post-processing functions have been properly investigated, the exact computation result cannot be obtained due to the distortion caused by wireless channels and additive noise in the practical fading environments. Therefore, there are several AirComp studies to reduce this error as much as possible by taking into account the practical environments.

In [6], the authors first discovered that linear source coding can be designed to reliably reconstruct a function over Gaussian MACs. Thereby, the signal transmission and computation process can be integrated. In [7], the authors mathematically verified that uncoded transmission, a simple linearly scaled version of sensor reading, can be optimal to minimize function distortion. On the other hand, AirComp is sensitive to synchronization errors due to simultaneous signal transmission. Hence, the authors in [4] proposed an analog function computation framework to be robust against synchronization errors by exploiting random sequence. In addition, [4] was implemented not only for simple linear functions, but also for various nomographic functions, and [8] verified the practice of the AirComp design ideas. Studies on massive inaccurate channel state estimation were investigated in [9,10]. In [11], the authors improved the performance of function computation by selecting sensors with large channel gain opportunistically. They discovered that, although the number of sensors is infinitely large, the computation rate does not converge to zero. In [12], the authors designed an adaptive analog function computation framework that each sensor adaptively sends its reading to for the AP to compute a desired function based on its nonuniform fading channel. In [13], a novel transceiver design was developed for the single-input multiple-output (SIMO) AirComp. The authors revealed the duality of the AirComp problem in [14]. They also showed a performance improvement based on the optimization theory. In [15], the authors considered implementing high-mobility multi-modal sensing and addressed issues corresponding to high hardware costs, power consumption, and huge channel state information (CSI) signaling overheads through a mixed-timescale optimization method. In [16,17], the authors researched a multiple-input multiple-output (MIMO) AirComp technique. They designed the beamformer through optimization, focusing on multi-function computation, not single-function computation. In [18], the method of computing multiple functions was proposed by applying non-orthogonal multiple access (NOMA) to AirComp. Moreover, various application fields to which AirComp is applied are being researched.

As previously mentioned, the conventional AirComp techniques in the MIMO sensor network mainly exploited multiple transmit antennae for spatial multiplexing to increase the data rate or computation rate. However, in practice, most sensor networks are static, requiring high energy efficiency and reliable AirComp techniques (e.g., smart home, factory automation) [19]. From this perspective, we propose a framework for single-function computation in MIMO IoT sensor networks to improve the reliability of the computation result by exploiting multi-transmit antennae for spatial diversity and formulate an optimization problem with an intuitive approach from the equation of mean squared error (MSE), which represents the performance of function computation. Specifically, when the receive beamforming vector is obtained through the optimization process at the AP, each wireless channel between each sensor and AP can be equivalently expressed as a vector, not a matrix. That is, the multiple-input single-output (MISO) channel appears equivalently. At this time, it is well-known that the maximum ratio transmission (MRT) technique is an optimal transmit beamforming technique in MISO systems [20]. Hence, to compute the function exploiting the wireless effective channel gain of multiple transmit antennae, we design the modified MRT technique which is slightly different to the conventional MRT due to the AirComp structure based on the transceiver design in [13]. In short, this structure is related to channel inversion and peak power constraint.

In addition, we introduce several methods to design the receive beamforming vector: receive antenna selection (RAS), semidefinite relaxation (SDR), successive convex approximation (SCA) and the randomization technique, which are well-known optimization techniques in the conventional MIMO communication systems [21,22]. After that, we elaborate on the formulation of optimization based on the MSE performance, and design the receive beamforming vector for the MIMO AirComp framework. Firstly, we consider a receive beamforming vector through the simplest method, RAS. Then, we perform the optimization process using the SDR method, which is a classic solution in transmit beamforming for multi-casting, as shown in [14]. Based on the constraints of the SDR method, we discuss the randomization method and the SCA algorithm, which are used to improve the performance of the SDR solution.

To the best of our knowledge, since the randomized SCA techniques have not been investigated in MIMO AirComp, we propose a framework which exploits the combination of the SCA algorithm with the randomization method (SCA randomization). This algorithm has a two-step process that uses randomization method in the SDR solution to generate an initial value and further refines it through the SCA algorithm [23]. The main contributions of this paper can be summarized as follows:

- We propose a MIMO AirComp framework for improving computational reliability rather than increasing the data rate or computation rate by using multiple transmit antennae in the static IoT sensor networks where IoT sensors and AP are operated fixedly. 
- We exploit MRT as a transmit beamforming technique to obtain the wireless effective channel gain of multiple transmit antennae in order to improve MSE performance. In particular, by applying this framework, conventional techniques can also be extended to MIMO systems.  
- We propose a novel two-step algorithm to design a receive beamforming vector that uses the randomization method in the initial SDR solution and further refine it through the SCA algorithm. Of course, each technique is well known in the MIMO communication systems. However, to the best of our knowledge, these techniques were first applied to the AirComp system in this paper.

The remainder of the paper is organized as follows. The system model with the transmit beamforming method is described in Section 2; in Section 3, we formulate the problem to design the receive beamforming vector in order to improve the performance in detail. Several techniques for designing receive beamforming vectors are derived in Section 3.1, Section 3.2, Section 3.3, Section 3.4, Section 3.5; the simulation results are shown in Section 4. Finally, conclusions are drawn in Section 5.

## 2. System Model

**Figure 1:**
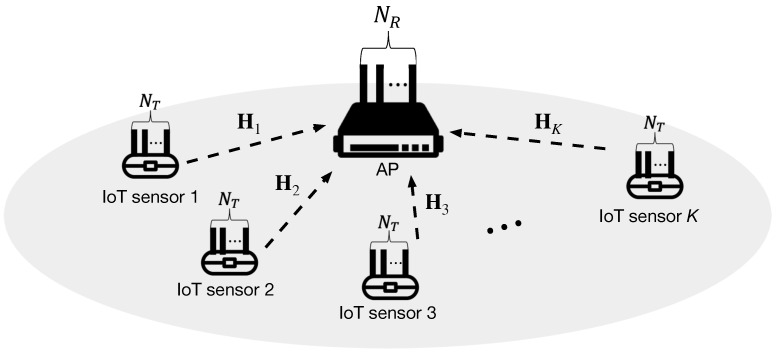
System model of the uplink multiple-input multiple-output (MIMO) wireless internet-of-things (IoT) sensor network.

We consider a MIMO over-the-air computation (AirComp) system, where *K* IoT sensors (STA) equipped with $N_{T}$ antennae, respectively, and an access point (AP) equipped with $N_{R}$ antennae exist, as illustrated in Figure 1. Herein, $H_{k}\in C^{N_{R}\times N_{T}}$ denotes the wireless channel coefficient matrix between the *k*-th STA and the AP. In this paper, we assume that each element of $H_{k}$ follows identically and independently distributed (i.i.d.) complex Gaussian distribution with zero mean and unit variance, i.e., 𝒞𝒩(0,1). It is also assumed that each STA has its own CSI, $H_{k}$, while the AP has the global CSI in the basic service set (BSS).

Let $\phi_{k}\left(\cdot \right)$ be the pre-processing function of the k(∈K={1,2,⋯,K})-th STA, $\psi \left(\cdot \right)$ be the post-processing function of the AP, and *f* be the computation result of the objective function, respectively. From the AirComp technique, the pre- and post-processing functions according to the objective function can be defined as shown in Table 1 [13]. In particular, *f* represents the exact computation result of the objective function of the BSS as follows:

(1) $$f\left(x_{1},x_{2},\cdots ,x_{K}\right)=\psi \;\left[\sum_{k=1}^{K}\phi \;\left(x_{k}\right)\right],$$

where $x_{k}\in R$ denotes the amplitude modulated (AM) symbol of the reading measured by STA *k*. In this paper, we only consider the arithmetic mean as the objective function due to its simplicity. That is, $f=\frac{1}{K}\sum_{k\in K}x_{k}$. Of course, other functions can be applied through a similar approach. For example, the geometric mean can be computed by applying a logarithmic function as a pre-processing function at each STA and an exponential function as a post-processing function at the AP. For simplicity of explanation, let it be assumed that there is no distortion caused by the wireless channel fading and additive white Gaussian noise (AWGN). When computing the geometric mean, the result of pre-processing at each STA, $\phi_{k}\left(x_{k}\right)=\log x_{k}$. Then, the superposition property can make the product of the sensing data of each STA by the logarithmic function. That is, the received signal, $y=\log\left(\Pi_{k\in K}x_{k}\right)$. Next, the post-processing exponential function, where the exponent is the same as the base of the logarithmic function, can be perfectly exploited to compute the objective function, the geometric mean, i.e., $f=\psi \left(y\right)=\exp\left(\log\left(\Pi_{k\in K}x_{k}\right)/K\right)={\left(\Pi_{k\in K}x_{k}\right)}^{1/K}$. However, in practice, the received signal will be distorted by the channel fading and thermal noise which cause the functional reliability to decrease. Hence, channel inversion is needed while satisfying the peak power constraint and the effect of a deteriorating performance due to noise can be suppressed.

The AM symbols of each STA are encoded with the modified MRT by exploiting the power control factor and receive beamforming vector to obtain the wireless channel gain of the multiple transmit antennae for the channel inversion. Considering the arithmetic mean as the simplest AirComp example of an objective function, the modified MRT signal of the STA *k*, $s_{k}\in C^{N_{T}}$, can be written as follows:

(2) $$s_{k}=\sqrt{\eta }\;\frac{{\left(w^{H}H_{k}\right)}^{H}}{∥w^{H}H_{k}{∥}^{2}}\;\phi_{k}\left(x_{k}\right)=\sqrt{\eta }\;\frac{{\left(w^{H}H_{k}\right)}^{H}}{∥w^{H}H_{k}{∥}^{2}}\;x_{k},$$

where η is a power control factor and $w\in C^{N_{R}}$ denotes a receive beamforming vector. Assuming the maximum transmit power of the STA is $P_{0}$ (i.e., $∥s_{k}{∥}^{2}\leq P_{0}$, ∀k), power control factor η can be defined as follows:

(3) $$\eta =P_{0}\min_{k}{∥w^{H}H_{k}∥}^{2}.$$

We assume that w and η are known at all STAs through the feedback process before transmission and that they were synchronized. Hence, all STAs can transmit modified MRT signals, (2), simultaneously via the same sub-carrier. Then, the received signal at the AP is given by

(4) $$y=\sum_{k=1}^{K}H_{k}s_{k}+n,$$

where $y\in C^{N_{R}}$ denotes the received signal vector at the AP, and $n\in C^{N_{R}}$ is the thermal noise vector with each element following i.i.d. $\mathrm{CN}(0,N_{0})$. From (4), the AP compensates for the wireless channel coefficients by exploiting the pre-designed receive beamforming vector as follows:

(5) $$\hat{g}=w^{H}y=\sqrt{\eta }\sum_{k=1}^{K}x_{k}+w^{H}n.$$

Finally, the arithmetic mean, the objective function of the BSS, can be computed through the scaling with known parameter η and the post-processing as follows:

(6) $$\hat{f}=\psi \left(\frac{\hat{g}}{\sqrt{\eta }}\right)=\frac{1}{K}\left(\sum_{k=1}^{K}x_{k}+\frac{w^{H}n}{\sqrt{\eta }}\right).$$

From (1) and (6), the distortion of $\hat{f}$ with respect to *f*, which quantified the AirComp performance, is estimated by the mean squared error (MSE), defined as follows:

(7) $$E\left[{\left|\hat{f}-f\right|}^{2}\right]=E\left[\frac{{∥w∥}^{2}N_{0}}{K^{2}P_{0}\min_{k}{∥w^{H}H_{k}∥}^{2}}\right].$$

Herein, we can intuitively see that the MSE performance of the MIMO AirComp is related to the signal-to-noise ratio (SNR) and the minimum effective channel gain among STAs regardless of the objective function, when the norm of the receive beamforming vector w is 1, i.e., ∥w∥=1.

## 3. Receive Beamforming Vector

In this section, we show the design process of the receive beamforming vector w and the optimization algorithm to minimize the MSE performance through w. From (7), it is clear that the MSE performance of AirComp is significantly related to the minimum effective channel gain of STAs. Thus, the optimization problem of the receive beamforming vector can be formulated as follows:

(8) $$\begin{array}{cc} \max_{w}\min_{k} & \;\;\;∥w^{H}H_{k}{∥}^{2}\\ s.\;t. & {\;\;\;∥w∥}^{2}=1. \end{array}$$

From this, we explain several methods of the receive beamforming vector design at the AP based on the receive antenna selection (RAS), semidefinite relaxation (SDR), randomization, successive convex approximation (SCA), and SCA with randomization.

### 3.1. Receive Antenna Selection (RAS)

First of all, the receive beamforming vector can be designed in a simple way by using the antenna selection method. In this method, the AP selects one antenna by considering MSE performance. Then, each STA performs the channel inversion using the CSI between itself and a selected antenna of the AP to transmit the modified MRT signal. The strategy of RAS to have as high an MSE performance as possible is chosen to select an antenna with the maximum minimum value of the effective channel coefficient. That is,

(9) $$m^{*}=\underset{m}{\arg}\max\min_{k}{∥h_{k,m}∥}^{2},$$

where $m^{*}$ denotes the selected antenna index and $h_{k,m}$ represents the CSI vector between STA *k* and the *m*-th antenna of the AP. Once the antenna is selected from (9), the MSE performance can be improved since the minimum effective channel gain would be increased. Finally, the receive beamforming vector $w={\left[w_{1},w_{2},\cdots ,w_{m},\cdots ,w_{N_{R}}\right]}^{T}$ of the RAS method can be defined as follows:

(10) $$w_{m}=\left\{\begin{array}{cc} 1, & \mathrm{if}\;m=m^{*}\\ 0, & \mathrm{otherwise} \end{array}\right..$$

RAS is the simplest method of designing a receive beamforming vector and shows improved MSE performance due to selection gain, but there are some limitations to the performance improvement since all receive antennae are not properly used (only one antenna is used for receive).

### 3.2. Semidefinite Relaxation (SDR)

From this subsection, the receive beamforming vector is designed based on the optimization theory. By introducing an auxiliary variable *c*, the problem (8) is the same, as follows:

(11a) $$\begin{array}{cc} \max & \;\;\;c\\ s.\;t. & \;\;\;c\leq ∥w^{H}H_{k}{∥}^{2},\;\;\forall k, \end{array}$$

(11b) $$\begin{array}{cc} \; & {\;\;\;∥w∥}^{2}=1. \end{array}$$

At this time, due to the non-convex set problem of (11), it is relaxed by using Semidefinite Programming (SDP), which contains a rank-1 constraint [24]. Then, we use the SDR method, which eliminates the rank-1 constraint. Assuming $W=ww^{H}$ and ${\hat{H}}_{k}=H_{k}H_{k}^{H}$, (11) can be transformed as follows:

(12a) $$\begin{array}{cc} \max & \;\;\;c\\ s.\;t. & \;\;\;c\leq \mathrm{trace}\left(W{\hat{H}}_{k}\right),\;\;\forall k, \end{array}$$

(12b) $$\begin{array}{cc} \; & \;\;\;W⪰0,\;\;\mathrm{trace}\left(W\right)=1. \end{array}$$

Herein, if rank $\left(W\right)=1$, the corresponding optimal receive beamforming vector, $w^{*}$, can be obtained as $W=w^{*}w^{*H}$. However, W is not always rank-1. If rank $\left(W\right)\neq 1$, W can be approximated by using eigenvalue decomposition with $W_{\mathrm{ap}}=\lambda_{1}v_{1}v_{1}^{H}$, where $\lambda_{1}$ is the maximum eigenvalue of W and $v_{1}$ is its corresponding eigenvector. In other words, the sub-optimal receive beamforming vector can be designed through the maximum eigenvalue approximation method, i.e., $w_{\mathrm{ap}}=\sqrt{\lambda_{1}}v_{1}$.

### 3.3. Randomization

The aforementioned SDR method-based solution cannot provide sufficient MSE performance in the design of the receive beamforming vector, since the solution of (12) is sub-optimal. Moreover, it is well-known that the performance of SDR-based solutions deteriorates as the dimension increases in the optimization process.

On the other hand, when solving quadratically constrained quadratic programs (QCQP), the randomization method has been widely used to develop the SDR solution. It is possible to devise a method to find a better solution by applying randomness to the rank-1 approximation. The randomization is performed on the solution derived from the SDR method as follows:

(13) $$w_{l}=w_{\mathrm{ap}}+\xi_{l},\;\mathrm{for}\;l\in \left\{1,2,\cdots ,L\right\},$$

where $\xi_{l}\in C^{N_{R}}$ is the *l*-th artificial vector consisting of the random numbers that follow the i.i.d. complex Gaussian distribution with zero-mean and $\sigma_{R}^{2}$-variance, i.e., $\mathrm{CN}(0,\sigma_{R}^{2})$, and *L* is the total number of randomization vectors. Due to the constraint in (7), the obtained randomization vectors should also be normalized. From the created *L* randomization vectors, the best random vector index, denoted as $l^{*}$, can be obtained as follows:

(14) $$l^{*}=\underset{l}{\arg}\max\min_{k}{∥w_{l}^{H}H_{k}∥}^{2}.$$

Then, the receive beamforming vector is designed in the following manner:

(15) $$w=\left\{\begin{array}{cc} w_{l^{*}}, & \mathrm{if}\;\;\min_{k}∥w_{l^{*}}^{H}H_{k}{∥}^{2}\geq \min_{k}∥w_{\mathrm{ap}}^{H}H_{k}{∥}^{2}\\ w_{\mathrm{ap}}, & \mathrm{otherwise} \end{array}\right..$$

This contains the following intuition: if the minimum effective channel gain of the beamforming vector obtained through randomization is greater than that obtained through the SDR method, a better MSE performance can be achieved in (7). In other words, among the *L* randomization vectors, the receive beamforming vector with a better performance than the existing SDR solution is replaced.

### 3.4. Successive Convex Approximation (SCA)

The SCA is one of the well-known methods to improve the performance of SDR-based solutions. To apply the SCA, we convert the problem (8) from the complex domain to the real domain using the following parameters [23]:

(16) w˜=RewTImwTT,

(17) H˜k=ReHkHkH−ImHkHkHImHkHkHReHkHkH,∀k.

Then, the problem (8) can be reformulated as follows:

(18) $$\begin{array}{cc} \max_{\tilde{w}}\min_{k} & \;\;\;{\tilde{w}}^{T}{\tilde{H}}_{k}\tilde{w}\\ s.\;t. & \;\;\;{∥\tilde{w}∥}^{2}=1. \end{array}$$

Moreover, the max–min problem can be transformed to the universal min–max problem by using ${\bar{H}}_{k}:=-{\tilde{H}}_{k},\;\forall k$, as follows:

(19) $$\begin{array}{cc} \min_{\tilde{w}}\max_{k} & \;\;\;{\tilde{w}}^{T}{\bar{H}}_{k}\tilde{w}\\ s.\;t. & \;\;\;{∥\tilde{w}∥}^{2}=1. \end{array}$$

Let us define ${\tilde{w}}^{T}{\bar{H}}_{k}\tilde{w}$ is $u_{k}\left(\tilde{w}\right)$, i.e., $u_{k}\left(\tilde{w}\right)≜{\tilde{w}}^{T}{\bar{H}}_{k}\tilde{w}$ for simplicity. Since $u_{k}\left(\tilde{w}\right)$ is a concave function, the tangent of the *n*-th point can be upper bounded as follows:

(20) $$\begin{array}{cc} u_{k}\left(\tilde{w}\right) & \leq \nabla u_{k}{\left({\tilde{w}}^{\left(n\right)}\right)}^{T}\left(\tilde{w}-{\tilde{w}}^{\left(n\right)}\right)+u_{k}\left({\tilde{w}}^{\left(n\right)}\right)\\ \; & ={\left(2{\bar{H}}_{k}{\tilde{w}}^{\left(n\right)}\right)}^{T}\tilde{w}-{\tilde{w}}^{(n)T}{\bar{H}}_{k}{\tilde{w}}^{\left(n\right)}. \end{array}$$

Using (20) and (12), the problem for each iteration can be reformulated as follows:

(21a) $$\begin{array}{cc} \min_{c\in R,\tilde{w}} & \;\;\;c\\ s.\;t. & \;\;\;{\left(2{\bar{H}}_{k}{\tilde{w}}^{\left(n\right)}\right)}^{T}\tilde{w}-{\tilde{w}}^{(n)T}{\bar{H}}_{k}{\tilde{w}}^{\left(n\right)}\leq c,\;\forall k, \end{array}$$

(21b) $$\begin{array}{cc} \; & \;\;\;∥\tilde{w}{∥}^{2}=1, \end{array}$$

where the initial solution ${\tilde{w}}^{\left(0\right)}$ is found through SDR. Then, the solution is iteratively updated and repeated until $∥{\tilde{w}}^{\left(n\right)}-{\tilde{w}}^{\left(n-1\right)}∥\leq \epsilon$. Finally, a solution representing the optimized receive beamforming vector ${\tilde{w}}^{*}$ is derived.

### 3.5. SCA with Randomization

The aforementioned SDR randomization and SCA were designed to improve the performance of the SDR by exploiting the SDR as an initial solution. From this perspective, we can design a two-step algorithm that combines two techniques: randomization and SCA. In other words, we exploit randomization in the SDR solution to generate an initial receive beamforming vector and further optimize it through the SCA algorithm. Specifically, we apply the randomization method in (15) and set the result as the initial solution for (21) instead of using the SDR solution. If the better solution is selected for the initial value of the SCA algorithm, it can quickly converge to the minimum point and find a better optimal point. The entire process of the algorithm is described in Algorithm 1.
**Algorithm 1** SCA with randomizationInitialize ϵ,n=1Solve (12) to obtain $w_{\mathrm{ap}}$               ▹ Performing SDR methodInitialize ${\tilde{w}}^{\left(0\right)}=w_{\mathrm{ap}}$**for**l=1,⋯,L**do**                  ▹ Performing randomization  Generate $w_{l}=w_{\mathrm{ap}}+\xi_{l}$, $w_{l}=w_{l}/∥w_{l}∥$  **if** $\min_{k}{∥w_{l}^{H}H_{k}∥}^{2}\geq \min_{k}∥{\tilde{w}}^{(0)H}H_{k}{∥}^{2}$ **then**    Update ${\tilde{w}}^{\left(0\right)}\leftarrow w_{l}$  **end if****end for**Set the initial solution ${\tilde{w}}^{\left(1\right)}$ by solving (21) with ${\tilde{w}}^{\left(0\right)}$**while**$∥{\tilde{w}}^{\left(n\right)}-{\tilde{w}}^{\left(n-1\right)}∥\geq \epsilon$**do**  Solve (21) to obtain $\tilde{w}$ using ${\tilde{w}}^{\left(n\right)}$        ▹ Performing SCA algorithm  Update ${\tilde{w}}^{\left(n+1\right)}\leftarrow \tilde{w}$  Update n←n+1**end while****return**$\tilde{w}$ as a solution of the algorithm

## 4. Simulation Results

**Figure 2:**
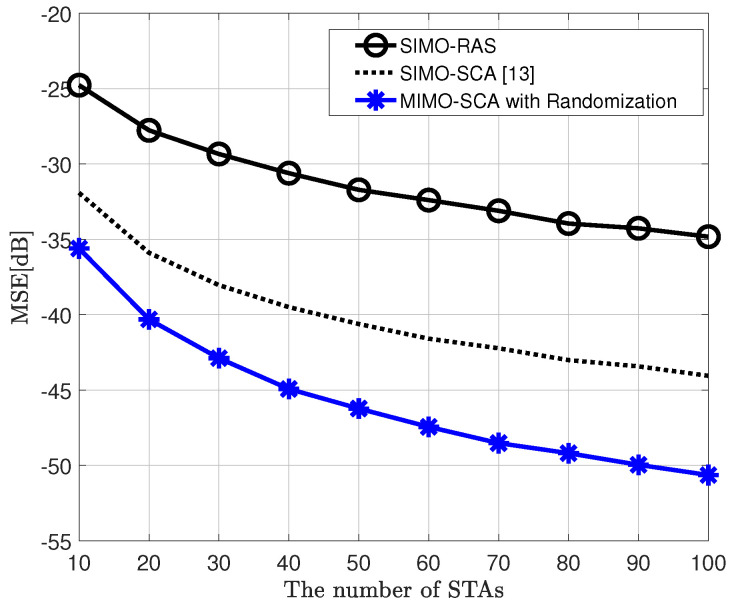
Mean squared error (MSE) performance of the proposed MIMO over-the-air computation (AirComp) framework and conventional single-input multiple-output (SIMO) AirComp, when signal-to-noise ratio (SNR) is 10 dB, objective function is arithmetic mean, and $N_{T}=2$ (in MIMO AirComp), $N_{R}=4$.

**Figure 3:**
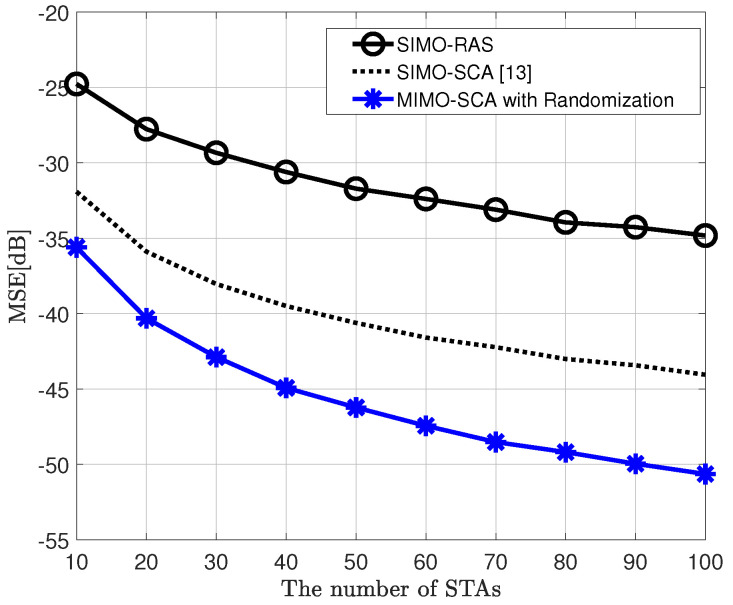
MSE performance of the proposed MIMO AirComp framework and conventional SIMO AirComp, when SNR is 10 dB, objective function is arithmetic mean, and $N_{T}=2$ (in MIMO AirComp), K=20.

Figure 2 shows the MSE performance versus the number of STAs, comparing the proposed MIMO AirComp framework, which is a two-step algorithm, and the conventional SIMO AirComp technique using the RAS or SCA algorithm for receive beamforming vector, when the objective function of the BSS is the arithmetic mean. Moreover, it is considered that the transmit SNR is 10 dB, $\sigma_{R}^{2}$ = 1 in the randomization method, and ϵ = 0.01 in the SCA algorithm. Herein, the MIMO-SCA with randomization is equipped with two transmit antennae. This figure shows that the proposed framework has improved MSE performance by up to 6 dB compared to the conventional SIMO AirComp with the SCA algorithm as the number of STAs increases even if only one transmit antenna is added. Furthermore, Figure 3 shows the MSE performance according to the number of receive antennae when the number of STAs is 20. As shown in Figure 2, the proposed MIMO AirComp framework outperforms the SIMO frameworks.

**Figure 4:**
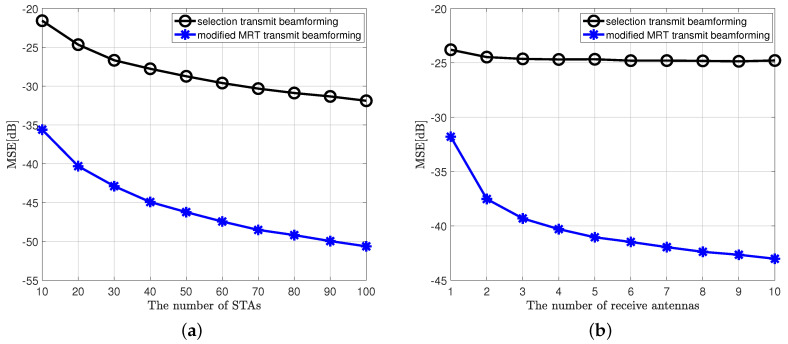
MSE performance of the proposed MIMO AirComp framework and other transmit beamforming technique, when SNR is 10 dB, objective function is arithmetic mean. (**a**) MSE performance when $N_{T}$ = 2, $N_{R}$ = 4. (**b**) MSE performance when $N_{T}$ = 2, *K* = 20.

Figure 4 shows the simulation results when considering the same parameters as Figure 2 and Figure 3, respectively. At this time, we numerically compared the proposed MIMO AirComp technique, exploiting the modified MRT and transmit selection beamforming, one of the other transmit beamforming techniques. As mentioned in Section 1, when the receive beamforming vector is obtained, each wireless channel between each sensor and AP can be expressed as a vector, i.e., the MISO channel appears equivalently. Moreover, it is well known that the MRT is an optimal transmit beamforming technique in MISO systems [20]. For this reason, modified MRT shows the best performance.

**Figure 5:**
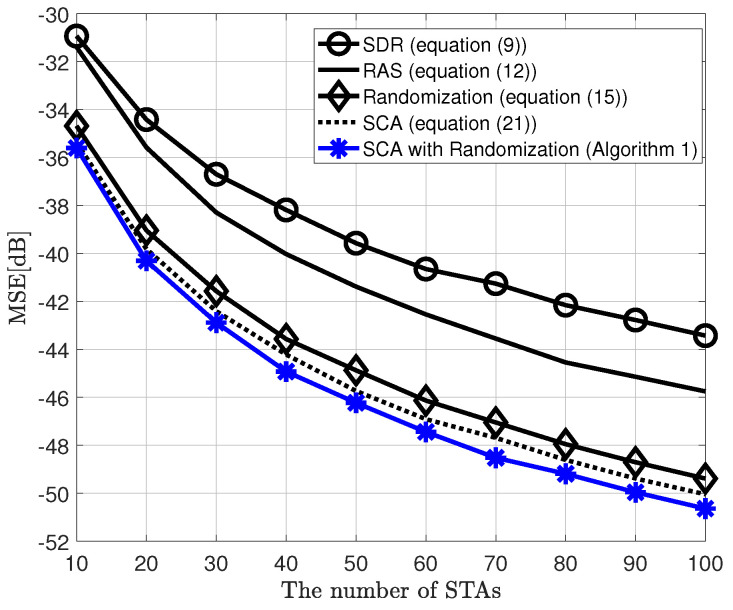
MSE performance of the proposed MIMO AirComp framework and other receive beamforming techniques, when SNR is 10 dB, objective function is arithmetic mean, and $N_{T}=2,N_{R}=4$.

Figure 5 shows the MSE performance versus the number of STAs of the proposed AirComp technique compared to other receive beamforming techniques. In Figure 5, the semidefinite relaxation (SDR) method described in (12) has a poor performance due to the rank-1 relaxation of the formulation. In addition, it is known that as the number of sensors increases, the degree of performance improvement gradually deteriorates (slope). Next, the receive antenna selection (RAS) method described in (9) is where the AP selects the best antenna with which the minimum channel gain of all IoT sensors was the maximum among the receive antennae. The randomization method that adds artificial random Gaussian noise into the SDR solution has been shown to have a considerably better performance than the two techniques above. As the number of sensors increases, the probability of a lower minimum effective channel gain among sensors is higher, and the probability of selecting the randomized solution is also higher. The successive convex approximation (SCA) algorithm has a slightly better performance than randomization.

On the other hand, the proposed two-step algorithm (SCA with randomization) shows the best performance. As the initial value of the SCA algorithm has been provided with a better value through the randomization process, it can converge the better solution from (21). When the number of sensors is small, it can be seen that the performance compared to conventional SCA is not significantly different. The reason is expected to be that when the dimension is low, the SDR-based solution is good enough, so the solution obtained through the randomization process may not be selected. However, as the number of IoT sensors increases, it is expected that the randomization method-based solution will be gradually selected and the MSE performance will improve accordingly.

**Figure 6:**
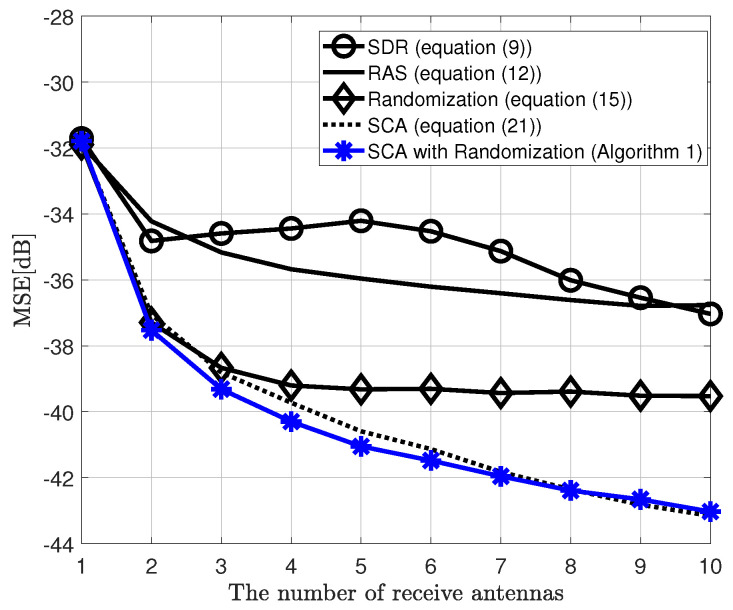
MSE performance of the proposed MIMO AirComp framework and other receive beamforming techniques, when SNR is 10 dB, objective function is arithmetic mean, and $N_{T}=2,K=20$.

Figure 6 shows the MSE performance versus the number of receiver antennae when the number of IoT sensors is 20, i.e., *K* = 20. First of all, the SDR method shows a poor performance due to the larger dimension, as previously mentioned. In particular, from three or more receive antennae, the MSE performance is distorted since the rank-1 approximation of the SDR is inaccurate [13]. On the other hand, the performance of the RAS method has gradually improved as the number of receive antennae increased thanks to the selection gain. However, the performance did not improve dramatically. The randomization method shows a better performance on a few antennae (when $N_{R}$ = 2 or 3), but it is saturated on a certain number of receive antennae (when $N_{R}$ = 4). This is because the performance of the SDR-based solution, which is the initial value of the randomization method, deteriorates as the number of receive antennae increases.

Finally, since the SCA and SCA with randomization exploit the SCA algorithm to refine the SDR solution in order to obtain a better optimized solution, they show an improved MSE performance in a larger number of receive antennae. In particular, the SCA with randomization method shows similar MSE performance with the SCA-based method as the number of receive antennae increases. The reason for this is expected to be that the randomization method-based solution, as previously mentioned, is independent for a certain number of receive antennae (when $N_{R}\geq 4$).

## 5. Conclusions

In this paper, we have investigated several novel over-the-air computation (AirComp) techniques with the optimized receive and transmit beamforming for uplinking multiple-input multiple-output (MIMO) wireless IoT sensor networks. We have exploited modified maximum ratio transmission (MRT) to maximize the wireless effective channel gain of the multiple transmit antennae. Moreover, we have proposed a receive beamforming vector design framework, a two-step algorithm based on successive convex approximation (SCA) with randomization to increase the computational reliability by improving the mean squared error (MSE) performance.

We have introduced the receive antenna selection (RAS) as the simplest method of designing the receive beamforming vector. Then, we have elaborated on the formulation of the optimization problem to obtain the optimized receive beamforming vector of the MIMO AirComp. At this time, due to the non-convexity of this optimization problem, we firstly adopted classical solution, the semidefinite relaxation (SDR) algorithm to relax the rank-1 constraint. However, the SDR solution is known to deteriorate when the dimension of a problem increases. Hence, we have adopted the randomization method that adds randomness into the SDR solution in order to improve the performance of MSE. Furthermore, we have introduced the SCA algorithm, which is another well-known method to improve the SDR solution.

Finally, we have combined two techniques so that the best performance is drawn out. The proposed two-step algorithm, SCA with randomization, uses randomization in the SDR solution to generate an initial approximation value in the first step; then, it is refined further through the SCA algorithm. The simulation results have shown that the proposed MIMO AirComp framework with two transmit antennae not only improved MSE performance by about 6dB compared to the conventional SIMO AirComp [13], but also shows the best performance when compared with other well-known optimization methods for AirComp. In addition, it has been shown that the proposed modified MRT outperforms other transmit beamforming techniques such as transmit selection beamforming.

Recently, AirComp techniques have been investigated in various applications. Among them, we believe non-orthogonal multiple access (NOMA), which is emerging as a multiple access technique thanks to its high spectral efficiency, is one of the techniques leveraging the AirComp framework [25,26]. Actually, as aforementioned in Section 1, AirComp with NOMA has been studied in wireless networks [18]. We leave NOMA-based AirComp, which combines the proposed MIMO AirComp with NOMA, to be investigated in further studies of massive IoT networks.

## Figures and Tables

**Table 1:** Examples of the objective functions [13].

Name	$\phi_{k}$	ψ	*f*
Arithmetic Mean	-	$\frac{\left(\cdot \right)}{K}$	$\frac{1}{K}\sum_{k\in K}x_{k}$
Weighted Sum	$a_{k}x_{k}$	-	$\sum_{k\in K}a_{k}x_{k}$
Geometric Mean	log(·)	exp((·)/K)	${\left(\Pi_{k\in K}x_{k}\right)}^{1/K}$
Polynomial	$a_{k}x_{k}^{\beta_{k}}$	-	$\sum_{k=1}^{K}a_{k}x_{k}^{\beta_{k}}$
Euclidean Norm	$x_{k}^{2}$	${\left(\cdot \right)}^{1/2}$	$\sqrt{\sum_{k=1}^{K}x_{k}^{2}}$

## Abbreviations

The following abbreviations are used in this manuscript:

AirComp	Over-the-air computation
MIMO	Multiple-input multiple-output
IoT	Internet-of-Things
STA	Station (IoT sensor)
AP	Access point
CSI	Channel state information
MSE	Mean squared error
RAS	Receive antenna selection
SDR	Semi-definite relaxation
SCA	Successive convex approximation
MRT	Maximum-ratio transmission
IDC	International Data Corporation
ZB	Zettabytes
SIMO	Single-input multiple-output
NOMA	Non-orthogonal multiple access
MISO	Multiple-input single-output
BSS	Basic service set
AM	Amplitude modulation
AWGN	Additive white Gaussian noise
SNR	Signal-to-noise ratio
QCQP	Quadratically constrained quadratic programs
